# Integrated molecular analysis of granulosa cells and follicular fluid extracellular vesicles reveals predictive biomarkers of oocyte maturation in the southern white rhinoceros

**DOI:** 10.3389/fcell.2026.1849851

**Published:** 2026-07-07

**Authors:** Ahmed Gad, Kristin Klohonatz, Nico G. Menjivar, Dawit Tesfaye, Elena Ruggeri

**Affiliations:** 1 Animal Reproduction and Biotechnology Laboratory (ARBL), College of Veterinary Medicine and Biomedical Sciences, Colorado State University, Fort Collins, CO, United States; 2 Department of Animal Production, Faculty of Agriculture, Cairo University, Giza, Egypt; 3 Center for Research on Reproduction and Women’s Health, Perelman School of Medicine, University of Pennsylvania, Philadelphia, PA, United States; 4 Stanford Fertility and Reproductive Health Services, Stanford Medicine Children’s Health, Sunnyvale, CA, United States; 5 Reproductive Sciences, Conservation Science Wildlife Health, San Diego Zoo Wildlife Alliance, Escondido, CA, United States

**Keywords:** assisted reproductive technologies, extracellular vesicles, miRNA, mRNA, oocytes, ovum pickup

## Abstract

**Introduction:**

Assisted reproductive technologies (ARTs) are an imperative conservation tool to rescue species from extinction. An essential step towards technological advancements and ARTs’ successes is identifying optimal *in vitro* culture conditions to mature oocytes, carry out fertilization, and culture embryos for future transfer. The role of follicular fluid-derived extracellular vesicles (FF-EVs) and granulosa cells (GC) in follicular development, oocyte maturation, and fertilization has been extensively studied for decades in human and domestic species models.

**Methods:**

This study aimed to identify transcriptomic differences in GC mRNA and miRNA expression, as well as miRNA cargo in FF-EVs in the southern white rhinoceros (SWR), and to integrate these datasets to uncover coordinated regulatory networks associated with oocytes that matured (with polarbody, PB) compared to those that failed to mature (without polar body, NoPB) following *in vitro* maturation.

**Results:**

Overall, in GCs, 1,117 transcripts (including both annotated and unannotated) were differentially expressed between groups. In GC-derived miRNAs, 11 were differentially expressed, all of which were upregulated in the PB group. In FF- EV samples, 15 miRNAs were identified as differentially expressed between the two groups. Comparison of predicted miRNA targets with the differentially expressed genes revealed multiple overlapping genes, suggesting a coordinated post-transcriptional regulation mechanism.

**Discussion:**

These miRNA-mRNA interactions are potential targets for further investigation, both as potential modulators to improve *in vitro* maturation outcomes and as biomarkers for predicting oocyte maturation potential in the SWR. In conclusion, the EV–mediated mRNA-miRNA signaling occurring during folliculogenesis in the SWR may offer critical insights into physiological reproductive conditions that contribute to oocyte maturation, ultimately informing the development of novel reproductive strategies to enhance ART outcomes in this species and others.

## 1 Introduction

Assisted reproductive technologies (ARTs) are vital conservation techniques employed to prevent species extinction. Advancements in biotechnologies applied in wildlife ARTs are a strategy that can enhance success in genetic rescue programs. As more is comprehended about unique physiological reproductive aspects in wildlife species, newer tools are developed and attempted to assist reproduction, mimicking the success of the application of these technologies in human and domestic species. An essential step towards technological advancements in ARTs’ success is identifying optimal *in vitro* culture conditions to mature oocytes, carry out fertilization, and culture embryos for future transfer. This goal can be achieved through a systematic study of the mechanisms of communication between the ovarian compartments and the follicular environment. The role of extracellular vesicles (EVs) and granulosa cells (GCs) in follicular development, oocyte maturation, and fertilization has been extensively studied in human and domestic species models. Granulosa cells, as prominent somatic follicular components, interact bidirectionally with the enclosed oocyte *via* EVs to regulate folliculogenesis and oocyte maturation through signaling pathways such as PI3K/AKT and MAPK (Ezzati and Izadpanah, 2025; Li et al., 2021). This framework directs interest in evaluating the EV-mediated molecular interactions and their impact on granulosa cell physiology, linked to oocyte meiotic competence acquisition. The mechanisms of communication between follicular somatic cells and the oocyte within the follicular microenvironment are critical areas of inquiry due to their fundamental role in follicular development, oocyte maturation, and fertility regulation (Hung et al., 2015; da Silveira et al., 2012; Andrade et al., 2017). Their role in modulating oocyte maturation, folliculogenesis, and early embryo development *via* miRNA-mediated regulation of core signaling networks could represent a major advancement in reproductive research for wildlife species where ART interventions are needed.

The southern white rhinoceros (SWR) has been previously used as an ambassador species to improve reproductive technologies in the closely related and functionally extinct northern white rhinoceros (NWR) and other rhinoceros species. Our previous study focused on the role of EVs in the bidirectional communication between the oocyte and follicular environment in the SWR (Gad et al., 2024), where we investigated for the first time the transport of regulatory molecules, such as microRNAs (miRNAs), and their potential role in regulating follicular growth and oocyte development within the follicular fluid (FF) during ovarian antral follicle development. However, the complete molecular fingerprint of the follicular microenvironment involving granulosa cells, oocytes, and FF-EVs is lacking in order to draw an integrated molecular network that governs follicular dynamics and oocyte developmental competence in the SWR.

Therefore, to further understand both the physiological and mechanistic aspects that involve oocyte meiotic competence acquisition and the ability of SWR oocytes to mature *in vitro*, we aimed to evaluate the mRNA-miRNA signatures from *in vivo* collected GCs and FF-EVs between oocytes that matured (extruded polar body = PB) and did not mature (no visible polar body = NoPB) after *in vitro* maturation. The cellular and extracellular transcriptome and miRNome data generated in the present study was integrated with our previously publsihed EV-mediated miRNA datasets (Gad et al., 2024) to uncover the coordinated mRNA-miRNA regulatory networks in the follicular microenvironment associated with oocyte competence in the SWR species.

## 2 Materials and methods

### 2.1 Ovum pickup (OPU), oocyte maturation, and biological material collection

**FIGURE 1:**
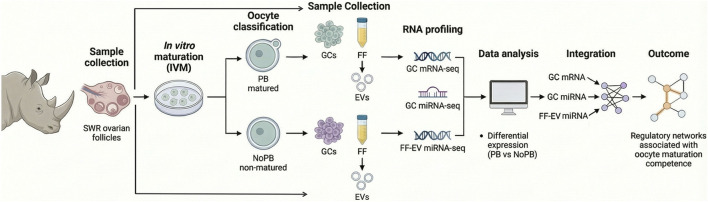
Experimental design. Ovarian follicles from southern white rhinoceros were subjected to *in vitro* maturation (IVM), and oocytes were classified as matured (PB) or non-matured (NoPB). Granulosa cells (GCs) and follicular fluid (FF) were isolated, and extracellular vesicles (EVs) were purified from FF. GC mRNA and miRNA, along with FF-EV miRNA, were profiled and integrated to identify regulatory networks associated with oocyte maturation competence.

All procedures, experiments, and methods were reviewed and approved by San Diego Zoo Wildlife Alliance’s Institutional Animal Care and Use Committee (SDZWA IACUC; protocol number 21-016). The entire experimental design can be found in Figure 1. The parous 12-year-old female SWR included in this study was confirmed by regular ultrasonographic examination to be free of reproductive pathologies. This female underwent ovarian stimulation before transrectal OPU as described previously (Ruggeri et al., 2024; Klohonatz et al., 2024; Ruggeri et al., 2023; Ruggeri et al., 2022). Briefly, the rhinoceros received synthetic chlormadinone acetate (CMA) at 3 mg/day for 18 days (days 0–17). Forty-eight hours after CMA withdrawal (day 19), she received 1.8 mg of deslorelin [gonadotropin-releasing hormone (GnRH) analog] followed by 2.5 mg of deslorelin 48 h later (day 22; 4.3 mg total) *via* intramuscular injection. OPU was performed on Day 24 as previously described (Ruggeri et al., 2024). The female was anesthetized using a combination of etorphine (3.8 mg), medetomidine (49.6 mg), azaperone (30.5 mg), and butorphanol (49.6 mg) administered intramuscularly *via* a remote drug delivery system. During initial positioning to facilitate intubation, propofol was administered intravenously (1,000 mg). Anesthesia was antagonized with 248 mg atipamezole and 191 mg naltrexone administered intramuscularly, resulting in a recovery without complications. Ovarian follicles were identified *via* ultrasound, then aspirated and flushed repeatedly with a warm (37 °C) flushing solution (Vigro) containing 12.5 I.U./mL of heparin. Follicular fluid and GCs from dominant follicles (18–26 mm) were collected and processed separately.

**FIGURE 2:**
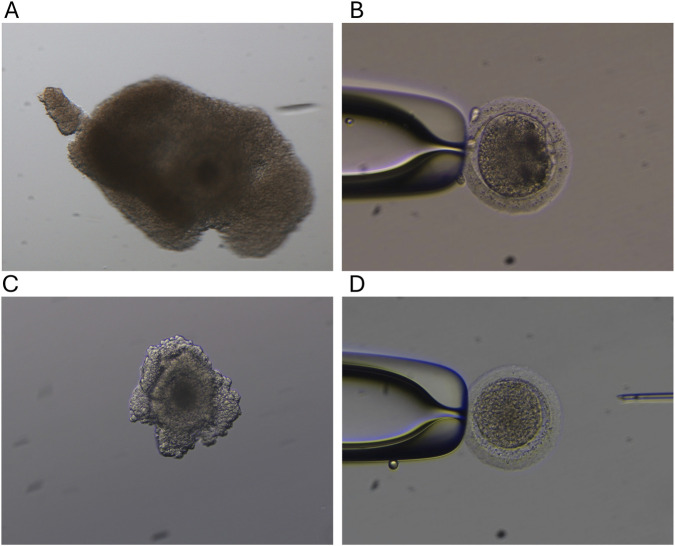
Representative images of PB and NoPB oocytes before and after *in vitro* maturation (IVM). **(A)** Cumulus oocyte complex (COC) at collection that extruded the first polar body after IVM. **(B)** Oocyte of A, after IVM was completed, showing polar body extrusion/mature, PB group. **(C)** COC at collection that did not extrude the first polar body after IVM. **(D)** Oocyte of C, after IVM was completed, showing no polar body extrusion/immature, NoPB group.

Follicle aspirates were maintained at 37 °C and immediately transported to the laboratory, where they were filtered through a 0.22-μm embryo filter (Professional Embryo Transfer Supply Inc.). Oocytes were retrieved from the collection fluid, evaluated, and placed into *in vitro* maturation (IVM) medium for 38 h at 37.5 °C, 6% CO_2_. IVM medium contained DMEM/F12, 10% FBS, ITS, gentamicin, LH, FSH, EGF, pyruvate, and IGF-1. Free-floating mural GC were collected and pipetted directly into RNAlater (Thermo Fisher Scientific, Waltham, MA) for 24 h at 4 °C. Multiple aliquots of GC from each follicle were then stored at −80 °C. After 38 h in IVM, oocytes were denuded by combination of exposure to hyaluronidase and manual stripping and maturation status was evaluated by the extrusion of the first polar body (Figure 2). Prior to storage, FFs were centrifuged at 300 *g* for 10 min, the supernatant was re-centrifuged at 2000 *g* for 10 min, and again at 16,500 x g for 30 min. The final supernatant was passed through a 0.20-μm sterile syringe filter, removing particles larger than 200 nm in diameter, and stored at −80 °C for further processing (Gad et al., 2024).

### 2.2 Follicular fluid extracellular vesicles isolation and characterization

Extracellular vesicles were isolated using a dual-step method combining ultracentrifugation and size exclusion chromatography as described previously (Gad et al., 2024). Briefly, 2 mL frozen–thawed FF samples were ultracentrifuged at 120,000×g for 70 min at 4 °C. The EV pellets were washed with DPBS and centrifuged again under the same conditions. The washed pellets were then resuspended in DPBS. To precipitate EVs, Exo-spin™ buffer was added to each sample and incubated overnight at 4 °C. The precipitated EVs were purified using Exo-spin™ mini columns according to the manufacturer’s instructions. Final 180 μL EV aliquots were stored at −80 °C for subsequent characterization and analysis.

*Nanoparticle Tracking Analysis (NTA):* The size distribution and particle concentration of isolated EVs were determined using a ZetaView® QUATT instrument (Particle Metrix). EV samples were diluted in sterile 1× DPBS and loaded into the system for measurement. Data were acquired at 11 different positions in scatter mode with a 488 nm laser. Particle concentration (particles/mL) was calculated using ZetaView software (v8.05.12 SP1) and adjusted according to the applied dilution factor.

*Transmission Electron Microscopy (TEM):* EV morphology and size were examined by transmission electron microscopy using a FEI Tecnai T12 Spirit microscope (FEI Company) operating at 100 kV and equipped with an AMT CCD camera. For preparation, 6–8 µL of isolated EV suspension was placed onto carbon-coated copper grids for 1–2 min, followed by negative staining with 2% aqueous uranyl acetate. After drying, samples were imaged, and approximately 15–20 representative micrographs were captured per sample for evaluation.

*Protein analysis:* Protein markers in frozen–thawed EV samples were evaluated using the JESS Simple Western™ system (ProteinSimple®, Bio-Techne). EV lysates (10 µL), prepared in RIPA buffer, were analyzed with the Assay Module for Protein Normalization (AM-PN001) using 25-capillary cartridges. EV markers (CD81, FLOT-1, and TSG101) were assessed, and the absence of the negative marker cytochrome C (CYCS) was confirmed. Data acquisition and analysis were performed using Compass for Simple Western software.

### 2.3 Extracellular vesicles RNA isolation, library preparation, and sequencing

The EV samples were used to isolate total RNA, including miRNAs, using a Norgen Exosomal RNA Isolation kit (Norgen, Canada). Genomic DNA contaminants were eliminated using on-column DNA digestion, and RNA concentration and size distribution were assessed using an Agilent 2,100 Bioanalyzer (Agilent Technologies, Santa Clara, CA, United States). Small-RNA libraries were prepared for next-generation sequencing with Illumina’s TruSeq Small RNA Library Prep Kit. Library quantity and quality were assessed using a Qubit 2.0 Fluorometer and an Agilent 2,100 Bioanalyzer. The libraries were then sequenced using a NovaSeq6000 instrument (Illumina, Inc., San Diego, CA, United States), as single-end reads (50 bases).

### 2.4 Granulosa cell RNA isolation, library preparation, and sequencing

For both RNA and miRNA isolation, the GCs were thawed, mixed in equal parts with cold PBS (Sigma Aldrich, St. Louis, MO), and centrifuged at 3,000 x g for 10 min. The pellet was resuspended in cold PBS. Samples were centrifuged again at 3,000 x g for 5 min to remove all remnants of RNAlater.

Total RNA for mRNA sequencing was isolated from GCs using the Arcturus PicoPure RNA Isolation Kit (Thermo Fisher Scientific, Waltham, MA) per the manufacturer’s instructions. Two follicles (biological replicates) were used for each group: polar body (PB; Figures 2A,C) and no polar body (NoPB; Figures 2B,D) outcome after *in vitro* maturation. Oocyte nuclear maturation completes primary meiotic division by extruding the first polar body; hence, the polar body presence indicates metaphase II arrest and meiotic maturation completion. For each biological replicate, there were two individual technical replicates from individual GC samples. Therefore, there was a total of n = 4 per group. Granulosa cells were incubated with the extraction buffer for 30 min before centrifugation to remove debris and extracellular material. The cell extract was incubated with ethanol, then bound to and washed on preconditioned purification columns. Total RNA was recovered in elution buffer and quantified using a Qubit 4 Fluorometer (Thermo Fischer Scientific, Waltham, MA). After quantification, the samples were evaluated on a 4,150 TapeStation System (Agilent Technologies, Santa Clara, CA) to determine RNA quality. Only samples with a RIN greater than seven were used for sequencing. Library preparation and RNA sequencing were performed at the University of California, San Diego Institute for Genomic Medicine Center. Following the manufacturer’s protocol, RNA-sequencing (cDNA) libraries were prepared using the Illumina TruSeq Stranded Total RNA Prep with Ribo-Zero Plus. A library was prepared for each sample using 50 ng of total RNA. Briefly, RNA was rRNA-depleted and fragmented, first- and second-strand cDNA synthesis was performed, and adapters were ligated. Each sample was amplified with a specific barcoded PCR primer for sample identification purposes. The prepared libraries were sequenced as 150-base pair paired-end reads on an Illumina NovaSeq 6,000 (Illumina, San Diego, CA).

For miRNA, two follicles (biological replicates) were used for the PB group, and one follicle was used for the NoPB group. For each biological replicate, there were two individual technical replicates from individual GC tubes. Therefore, there was a total of n = 4 for PB and n = 2 for NoPB. Due to technical challenges related to limited amounts of sample, only two technical replicates/one biological replicate were available for miRNA isolation for the NoPB group. Total RNA, including miRNA, was isolated using the Qiagen miRNeasy Micro Kit (Qiagen, Germantown, MD) per the manufacturer’s instructions. Briefly, GCs were incubated with Qiazol lysis reagent and vortexed to homogenize the cells. After 5 min, chloroform was added to the sample and centrifuged at 10,000 x g for 15 min to separate RNA and protein. The RNA phase was added to an elution column and eluted in water after multiple washes. The samples were evaluated on a 4,150 TapeStation System (Agilent Technologies, Santa Clara, CA) to determine RNA quantity and quality. Only samples with a RIN greater than 9.5 were used for sequencing. Small-RNA library preparation and sequencing were completed by Novogene Corporation. The library preparation kit was SMARTer smRNA-Seq Library Preparation Kit (Takara Bio) per manufacturer’s instructions. This preparation method resulted in an average final library size of 300 bp. Illumina 8-nt-dual-indices were used for multiplexing. Library quantity and quality were assessed by High Sensitivity RNA Tapestation (Agilent Technologies). Samples were pooled and sequenced on an Illumina NovaSeq X Plus 10B sequencer as 150 base pair read length in paired-end mode.

### 2.5 mRNA sequencing data analysis

Granulosa cell mRNAseq data analysis was performed on the Galaxy web platform using the public server usegalaxy.org (Afgan et al., 2018). Initial assessment of sequence quality was performed by FastQC, and the sequences were aggregated for comparison by MultiQC (Andrews, 2010; Ewels et al., 2016). The adapter sequences and reads with quality scores below the quality threshold of 25 and with fewer than 20 base pairs were removed from analysis *via* Trimmomatic (Bolger et al., 2014). HISAT2 was used to align reads to the northern white rhinoceros (NWR) genome CerSimCot1.0 (GCA_021442165.1) (Kim et al., 2019; Wang et al., 2025). The southern white rhinoceros genome (CerSimSim1.0; GCA_000283155.1) was not used for this analysis due to large gaps in the sequencing and extremely limited annotation. Transcript assembly and quantification for both annotated and unannotated transcripts were initially performed with Stringtie, and each animal sample was analyzed individually (Pertea et al., 2015). The newly released annotation for CerSimCot1.0 was used for alignment (Ruggeri et al., 2026). The resulting transcripts for individual samples were merged to create a final annotation file representing the intersection and union of all samples (Pertea et al., 2015). Stringtie was subsequently applied to the complete merged annotation to generate transcript read counts. Transcript counts were exported from Galaxy and imported into RStudio for statistical analysis (R Core Team, 2013). Transcript read counts were analyzed between each follicle type utilizing DESeq2 within R (Love et al., 2014). The average expression value for replicates within a group was ≥3, except in the cases where the expression value was 0 for the replicates within a group. When not expressed in a group, the expression value for all replicates was 0. The data were normalized internally using the median ratio method of DESeq2. Benjamini–Hochberg false discovery rate adjustment was used. Significance was assessed at *P* ≤ 0.05. Visualizations were prepared by Venny (Oliveros, 2007) and R Studio (R Core Team, 2013). Biological pathway and KEGG pathway analyses were completed with the Database for Annotation, Visualization, and Integrated Discovery (DAVID) (Sherman et al., 2022; Huang et al., 2009). The raw data and processed files were uploaded to the Gene Expression Omnibus under accession number GSE300824.

### 2.6 Small RNA sequencing data analysis

For GCs miRNA, due to limited sample availability, there was an n = 2 for the NoPB group and n = 4 in the PB group. This sample size difference was accounted for in the statistical analysis. Small RNA-seq data were processed and analyzed using the Galaxy platform. Raw FASTQ files were first subjected to quality control and adapter trimming using FASTp, with a minimum Phred quality score of 20, a required minimum read length of 15 nucleotides, and a maximum read length of 35 nucleotides. Filtered reads were processed using the miRDeep2 pipeline within the Galaxy platform. Briefly, reads were first subjected to the miRDeep2 Mapper module for collapsing and genome alignment, discarding reads shorter than 17 nucleotides. Both reference-based and genome-guided miRNA identification were then performed using miRDeep2. For known miRNA quantification, the miRDeep2 Quantifier module was used with miRBase release 22.1 as the reference database, annotating reads against equine, bovine, and human precursor and mature miRNAs with up to one mismatch allowed. In parallel, novel miRNA prediction was conducted using the miRDeep2 core algorithm based on read alignment signatures and characteristic hairpin secondary structures mapped to the NWR genome assembly CerSimCot1.0 (GCA_021442165.1). A known miRNA detected in at least one sample was considered expressed. Putative novel miRNA candidates were filtered to retain high-confidence predictions based on the following criteria: miRDeep2 score ≥10, total read count ≥10, significant randfold p-value, and detection in at least two samples within each group. Due to the sample-specific assignment of provisional identifiers by miRDeep2, novel miRNAs were harmonized across samples and experimental groups based on identical mature sequences and genomic coordinates, and unified identifiers were assigned to represent each unique miRNA.

Differential expression analysis was conducted using DESeq2 within Galaxy. miRNAs with a fold change (FC) ≥ 2 and a p-value <0.05 were considered differentially expressed (DE-miRNAs). The raw data and processed files were uploaded to the Gene Expression Omnibus under accession number GSE326340.

To comprehensively identify target genes, human DE-miRNAs and the human homologs of equine and bovine DE-miRNAs were used. Validated targets were retrieved from miRTarBase (v9.0), and predicted targets were obtained from TargetScan (Release 8.0) using a cumulative weighted context++ score threshold of < −0.4. Functional enrichment and ontological classification analyses were performed using the DAVID bioinformatics resource (https://david.abcc.ncifcrf.gov/). Significantly enriched pathways and biological processes were identified from the KEGG pathway database (Kanehisa et al., 2025) and the GO Biological Process (GOTERM_BP_DIRECT) annotation set, respectively. Interaction networks of DE-miRNAs across different comparisons were constructed using Cytoscape (Shannon et al., 2003). Visualizations were prepared by Venny (Oliveros, 2007) and R Studio (R Core Team, 2013).

## 3 Results

### 3.1 Gene expression profiles in granulosa cells

**FIGURE 3:**
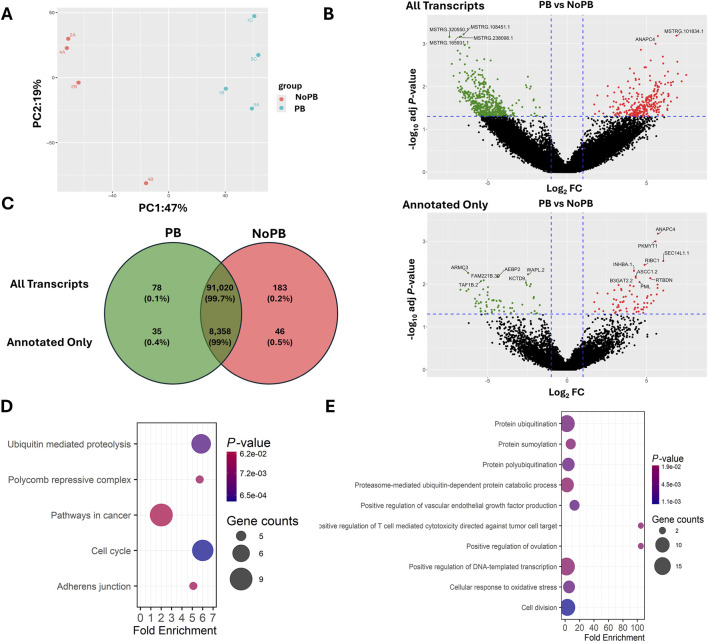
Gene expression profiles in granulosa cells. **(A)** PCA plot for all the samples analyzed. **(B)** Volcano plots representing both the significantly up- (red) and downregulated (green) genes (P < 0.05; |FC| > 1) in the PB compared to the NoPB group. **(C)** A Venn diagram representing the commonly and exclusively expressed genes (total and only annotated). Bubble plots representing the **(D)** KEGG pathways and **(E)** biological processes of differentially expressed genes from the granulosa cells. The P-value and gene count are represented by color and bubble size, respectively.

Prior to downstream analysis, principal component analysis (PCA) was performed to assess sample variability and group separation between GCs derived from follicles yielding oocytes that matured (PB) and those that did not (NoPB). The PCA results revealed a tight clustering of biological replicates within each group and a clear separation between PB and NoPB samples, indicating high within-group consistency and distinct transcriptional profiles between conditions (Figure 3A). The total number of detected transcripts, including the annotated ones in both groups is summarized in Figure 3C. Overall, there were 1,117 differentially expressed genes (DEGs) between the two groups, including both annotated (207) and unannotated (910) genes (Figure 3B). For the annotated genes, four were represented by multiple different transcripts. Further analysis revealed that of the 207 DEGs, 100 were upregulated in the PB group, with 35 being expressed exclusively in that group. Table 1 contains the top 10 DEGs based on p-value (where all genes were expressed exclusively in either PB or NoPB) for both annotated and unannotated genes, and Table 2 contains the top 10 DEGs based on fold change for only the annotated genes. In contrast, 107 annotated DEGs were upregulated in the NoPB group, with 46 being exclusively expressed in that group. For the unannotated DE transcripts (910), 265 were upregulated in the PB group. Of these 265, 43 genes were exclusively expressed in the PB group. In the NoPB group, 645 were DE, and 138 were exclusive to this group. The annotated and unannotated DEGs are summarized in Supplementary Table S1. The biological processes and KEGG pathway analysis revealed that most DEGs were involved in ubiquitin-mediated proteolysis, polycomb repressive complex, pathways in cancer, cell cycle, and adherence junctions (Figure 3D). The most common biological processes were protein ubiquitination, protein sumoylation, protein polyubiquitination, and proteosome-mediated ubiquitin-dependent protein catabolic process (Figure 3E).

**TABLE 1:** Top ten differentially expressed genes in granulosa cells sorted by p-value.

Gene ID	P-value	PB expression level	NoPB expression level
*CABIN1*	1.22E-15	1,446.3	0.0
*DST*	3.57E-14	721.0	0.0
*ATF4*	4.50E-13	630.0	0.0
*PTCHD4*	1.28E-12	0.0	619.7
*NR3C1*	3.56E-12	0.0	579.0
*MSTRG.442643.84*	5.34E-12	0.0	689.0
*PRRC2B*	5.88E-12	611.5	0.0
*MBNL2*	5.88E-12	0.0	632.0
*ENTPD6*	1.23E-11	794.3	0.0
*EPB41L5*	1.45E-11	0.0	539.0

The gene represented by MSTRG is unannotated. There are no fold changes listed because expression for each of these genes was exclusively in either PB or NoPB.

**TABLE 2:** Top ten annotated differentially expressed genes in granulosa cells sorted by fold change.

Gene ID	P-value	log2(FC)	PB expression level	NoPB expression level
*ARMC8*	1.34E-02	−6.71	1.0	104.5
*CSMD3*	1.45E-02	−6.33	1.5	120.8
*ARMC3*	5.46E-03	−6.22	2.8	205.3
*FHL5*	1.32E-02	−6.21	2.0	147.7
*SEC14L1*	2.87E-03	6.05	82.7	1.3
*PA2G4*	1.41E-02	6.05	148.8	2.3
*RRP15*	2.20E-02	−5.80	3.0	167.0
*NAIF1*	3.31E-02	−5.77	1.8	95.5
*KLHL18*	2.80E-02	−5.75	1.3	67.3
*ANAPC4*	6.65E-04	5.71	511.3	9.8

A positive fold change indicates higher expression in the PB group.

### 3.2 miRNA expression profiles in granulosa cells

**FIGURE 4:**
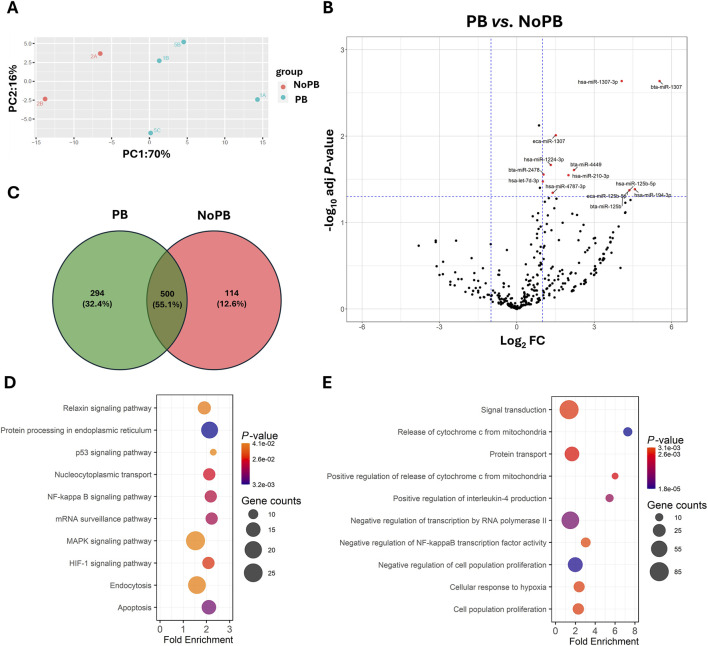
Functional enrichment analysis of miRNA target genes in granulosa cells. miRNA expression profiles in granulosa cells. **(A)** PCA plot for all the samples analyzed. **(B)** Volcano plot highlighting the significantly differentially expressed miRNAs (P < 0.05); |FC| > 1). **(C)** A Venn diagram representing the commonly and exclusively expressed miRNAs in PB and NoPB. Bubble plots illustrating enriched **(D)** KEGG pathways and **(E)** Gene Ontology (GO) biological processes associated with predicted target genes of miRNAs. Bubble size represents gene count, and color indicates the degree of enrichment.

Summary data for the miRNA expression profiles in GCs can be found in Figure 4. Biological replicates within each group clustered together indicated in the PCA (Figure 4A). Overall, there were a total of 500 commonly expressed miRNAs between the PB and NoPB groups (Figure 4C). Differential expression analysis revealed a total of 11 DE-miRNAs all of which are upregulated in GCs from the PB compared to the NoPB group (Figure 4B; Table 3). Target gene prediction identified a total of 1,156 genes targeted by the 11 DE-miRNAs. These genes are involved in several pathways, including MAPK signaling, apoptosis, endocytosis, and protein processing in the endoplasmic reticulum (Figure 4D), and biological processes, including signal transduction and negative regulation of transcription by RNA polymerase II (Figure 4E).

**TABLE 3:** Differentially expressed miRNA in granulosa cells collected from the PB compared to the NoPB group.

Species	miRNA ID	log2(FC)	P-value
bta-	**miR-1307**	5.53	0.002305
hsa-	miR-1307-3p	5.53	0.002305
hsa-	miR-194-3p	4.58	0.041133
bta-	miR-125b	4.36	0.042177
eca-	**miR-125b-5p**	4.36	0.042177
hsa-	**miR-125b-5p**	4.36	0.042177
bta-	miR-4449	2.21	0.024649
hsa-	miR-210-3p	2.00	0.028224
eca-	**miR-1307**	1.51	0.009764
hsa-	miR-4787-3p	1.39	0.045215
hsa-	miR-1224-3p	1.31	0.02152
bta-	miR-2478	1.04	0.02779
hsa-	let-7d-3p	1.01	0.033387

Species represented are: bta-bovine, hsa-human, and eca-equine. miRNAs that are bolded represent those that have been identified in multiple species. A positive fold change indicates higher expression in the PB group.

Genome-guided miRNA analysis using miRDeep2 enabled the identification of putative novel miRNAs in GCs. Following filtering for high-confidence candidates, a total of 15 novel miRNAs were detected in the PB group (Supplementary Table S2), while 5 novel miRNAs were identified in the NoPB group (Supplementary Table S3). Among these, 3 novel miRNAs were commonly detected in both groups, whereas the remaining candidates were group-specific. These findings suggest the presence of distinct and shared novel miRNA signatures associated with oocyte maturation status.

### 3.3 Molecular and morphological characterization of follicular fluid–derived extracellular vesicles (FF-EVs)

**FIGURE 5:**
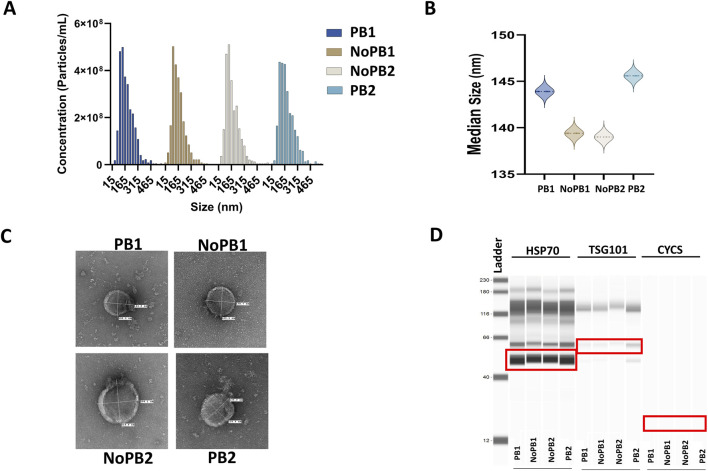
Characterization of follicular fluid–derived extracellular vesicles (FF-EVs). **(A)** Nanoparticle tracking analysis (NTA) showing EV size distribution and particle concentration across samples. **(B)** Median EV size in PB and NoPB groups. **(C)** Representative transmission electron microscopy (TEM) images of EVs from each sample, demonstrating characteristic cup-shaped morphology and vesicle diameter. **(D)** Protein characterization by Jess Simple Western analysis confirming the presence of EV markers HSP70 and TSG101 and the absence of the mitochondrial protein cytochrome c (CYCS).

Comprehensive characterization of FF-EVs confirmed their consistency, integrity, and purity across all samples. Particle size distribution and concentration were comparable among groups (Figure 5A), with PB samples (F1 and F5) showing a slight, non-significant increase in median vesicle size relative to NoPB samples (F2 and F4). Transmission electron microscopy revealed vesicles with the characteristic cup-shaped morphology in all samples (Figure 5C). Molecular profiling using the Jess Simple Western platform confirmed the presence of canonical EV markers, HSP70 and TSG101, in all preparations, while the mitochondrial protein cytochrome c (CYCS) was not detected, indicating the absence of cellular content contamination (Figure 5D).

### 3.4 miRNA expression profiles in follicular fluid extracellular vesicles (FF-EVs)

**FIGURE 6:**
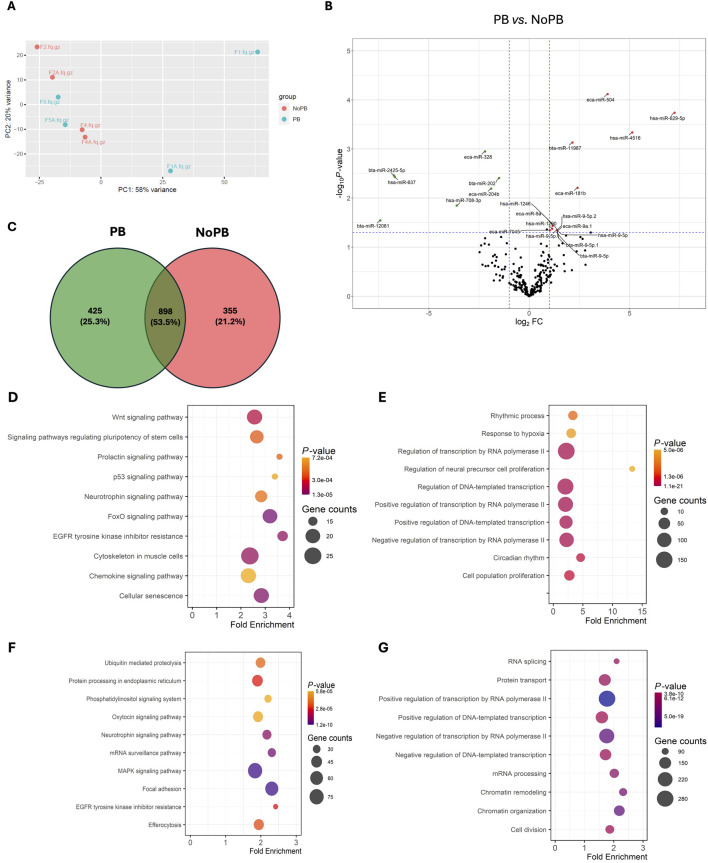
Functional enrichment analysis of target genes of differentially expressed miRNAs in follicular fluid–derived extracellular vesicles (FF-EVs). miRNA expression profiles in follicular fluid extracellular vesicles. **(A)** PCA plot for all the samples analyzed. **(B)** Volcano plot highlighting the significantly differentially expressed miRNAs (P < 0.05); |FC| > 1). **(C)** A Venn diagram representing the commonly and exclusively expressed miRNAs in the PB and NoPB groups. Bubble plots showing enriched **(D,F)** KEGG pathways and **(E,G)** Gene Ontology (GO) biological processes associated with predicted target genes of DE miRNAs. Panels **(D,E)** represent targets of miRNAs upregulated in the PB group, whereas panels **(F**,**G)** correspond to targets of miRNAs upregulated in the NoPB group (i.e., downregulated in PB). Bubble size indicates gene count, and color represents the degree of enrichment.

In contrast to mRNA and miRNA expression profiles in GCs, FF-EVs miRNA profiles showed limited separation between samples, with no clear clustering observed in the PCA plot. (Figure 6A). A total of 898 miRNAs were commonly detected across both groups (Figure 6C), among which 15 exhibited significant differential expression (Figure 6B). Nine miRNAs were upregulated (miR-629-5p, miR-4516, miR-504, miR-181b, miR-11987, miR-9-5p, miR-1246, miR-1290, and miR-7045) and six were downregulated (miR-202, miR-204b, miR-328, miR-637, miR-2425-5p, and miR-12061) in the PB compared to the NoPB group (Figure 6B; Table 4). Notably, no differentially expressed miRNAs were conserved across multiple species in this study. The DE-miRNAs were then used to predict target genes. Upregulated miRNAs in the FF-EVs from the PB group were predicted to target 1,110 genes, whereas those upregulated in the NoPB group targeted 2,780 genes.

**TABLE 4:** Differentially expressed miRNA in follicular fluid extracellular vesicles.

Species	miRNA ID	log2(FC)	P-value
hsa-	miR-629-5p	7.24	0.000182
hsa-	miR-4516	5.14	0.000458
eca-	miR-504	3.91	7.63E-05
eca-	miR-181b	2.41	0.006151
bta-	miR-11987	2.15	0.000739
hsa-	miR-9-5p	1.38	0.045276
hsa-	miR-1246	1.16	0.03702
hsa-	miR-1290	1.14	0.042225
eca-	miR-7045	1.04	0.045092
bta-	miR-202	−1.50	0.003902
eca-	miR-204b	−1.89	0.006416
eca-	miR-328	−2.20	0.001122
hsa-	miR-637	−6.70	0.003695
bta-	miR-2425-5p	−6.74	0.00347
bta-	miR-12061	−7.42	0.028471

Species represented are: bta-bovine, hsa-human, and eca-equine. A positive fold change indicates expression was higher in the PB group.

Functional enrichment analysis of the predicted target genes for DE-miRNAs from FF-EVs was performed using KEGG pathways and Gene Ontology (GO) biological processes (Figure 6). Among the targets of the miRNAs upregulated in the PB group, the most enriched KEGG pathways included Wnt signaling, signaling pathways regulating pluripotency of stem cells, and the prolactin signaling pathway. In contrast, the targets of the miRNAs downregulated in the PB group (i.e., upregulated in NoPB) were primarily enriched in ubiquitin-mediated proteolysis, protein processing in the endoplasmic reticulum, and the phosphatidylinositol signaling system (Figures 6D,F).

Gene Ontology analysis further revealed distinct biological processes between groups. Target genes of miRNAs upregulated in PB were predominantly associated with rhythmic processes, response to hypoxia, and regulation of transcription by RNA polymerase II. Conversely, targets of miRNAs upregulated in the NoPB group were enriched in RNA splicing, protein transport, and positive regulation of transcription by RNA polymerase II (Figures 6E,G).

Genome-guided miRNA analysis using miRDeep2 identified a set of high-confidence novel miRNA candidates in FF-EVs. A total of 30 and 28 novel miRNAs were detected in the PB and NoPB groups, respectively (Supplementary Table S4, S5). Among these, 15 novel miRNAs were commonly detected in both groups, while the remaining candidates were group-specific. These results indicate both shared and distinct novel miRNA signatures in FF-EVs associated with oocyte maturation status.

### 3.5 Integrated analysis of DEGs and DE-miRNAs

**FIGURE 7:**
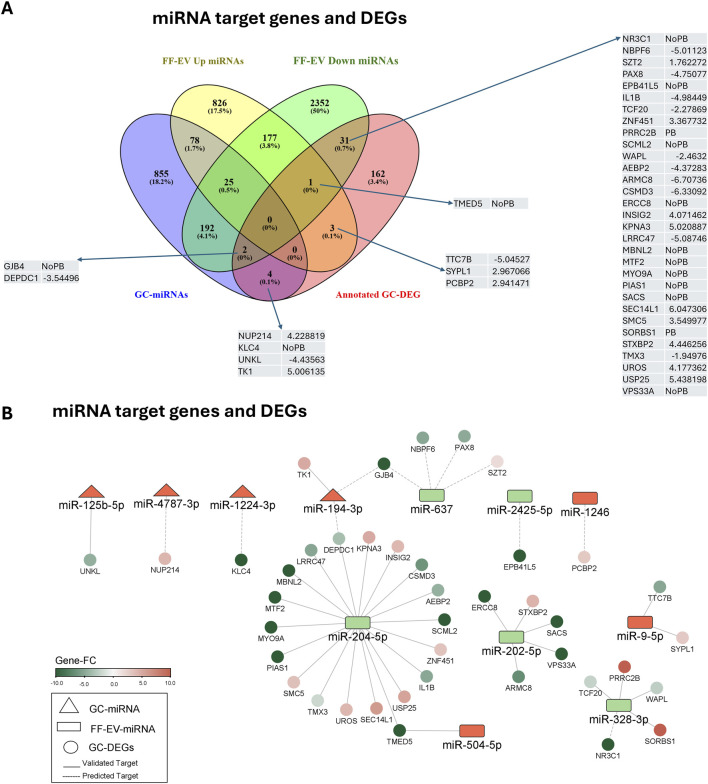
**(A)** Visual representation of the crossover between the granulosa cells (GC) miRNAs and follicular fluid extracelluar vesicles’ (FF-EVs) gene targets and the differentially expressed genes in the GCs. Integrated miRNA–mRNA interaction network in granulosa cells and follicular fluid–derived extracellular vesicles (FF-EVs). **(B)** Network visualization of the differentially expressed (DE) miRNAs from granulosa cells (triangles) and FF-EVs (rectangles) and their predicted (dashed lines) or validated (solid lines) target genes (circles) overlapped with the GC DEGs. Node color represents gene expression fold change (green = downregulated; red = upregulated).

To further investigate regulatory relationships between DE-mRNA and miRNA, we assessed the overlap between DEGs identified in GC mRNA and the predicted targets of DE-miRNAs in both GCs and FF-EVs (Figure 7A). Among the 100 DEGs upregulated in the PB group, two genes (*NUP214* and *TK1*) were identified as predicted targets of DE-miRNAs in the GCs. Among the 107 DEGs upregulated in the NoPB group, four genes (*DEPDC1, GJB4, KLC4,* and *UNKL*) were identified as targets of the DE-miRNAs in GCs. When comparing the DEGs to the targets of the FF-EVs, 13 were in common with the FF-EVs that were upregulated in PB (*SZT2, PCPB2, SYPL2, ZNF451, SMC5, INSIG2, UROS, STXBP2, KPNA3, USP25, PRRC2B, SORBS1,* and *SEC14L1*) and 24 were in common with the FF-EVs that were downregulated in PB (*ARMC8, CSMD3, LRRC47, TTC7B, NBPF6, IL1B, PAX8, AEBP2, DEPDC1, WAPL, TCF20, TMX3, NR3C1, EPB41L5, SCML2, ERCC8, MBNL2, MTF2, MYO9A, PIAS1, SACS, VPS33A, GJB4,* and *TMED5*). Both the FF-EVs and the GC miRNAs targeted two DE-mRNA (*GJB4* and *DEPDC1*), both of which were upregulated in the NoPB group. There were four targets of the GC miRNAs that were not targets of FF-EVs (*NUP214, KLC4, UNKL,* and *TK1*). Network analysis integrating target genes of DE-miRNAs from GCs and FF-EVs with GC DEGs genes highlighted complex regulatory interactions (Figure 7B). Notably, miR-204-5p emerged as a hub miRNA, targeting many DEGs, including genes involved in transcriptional regulation and cellular signaling (e.g., *MTF2, MYO9A, PIAS1,* and *SCML2*). Additional miRNAs, such as miR-202-5p and miR-328-3p, were also found to interact with multiple targets. Two DEGs (*DEPDC1*and *GJB4*) were targeted by miRNAs derived from both GC and FF-EV sources, supporting shared regulatory mechanisms between intracellular and extracellular compartments.

## 4 Discussion

Recent advances in ARTs in the white rhinoceros (Hildebrandt et al., 2023; Gad et al., 2024; Klohonatz et al., 2024; Ruggeri et al., 2024; Ruggeri et al., 2023; Ruggeri et al., 2022) have expanded our understanding of reproductive biology and hold significant potential to improve *in vitro* ART methodologies for this species. Techniques including ovum pickup (OPU), *in vitro* oocyte maturation, fertilization, and embryo production (Hildebrandt et al., 2023; Hildebrandt et al., 2021) have now been implemented, offering promising avenues for restoring the genetic diversity required to sustain viable populations in endangered species like the SWR. However, given the limited number of research groups engaged in this complex field, there remains a critical need for innovative strategies to enhance the efficiency and success of these technologies. In particular, the molecular mechanisms underlying follicular dynamics and ovarian function in rhinoceroses remain largely unexplored, underscoring the importance of developing advancements adapted from established model systems.

Prior work from our group has identified the transcriptomic signatures of granulosa cells from oocytes collected *in vivo* (Klohonatz et al., 2024; Ruggeri et al., 2024; Ruggeri et al., 2023). Extracellular vesicles (EVs) play a central role in mediating molecular communication between the developing oocyte and surrounding maternal tissues, with microRNAs (miRNAs) acting as key regulators of follicular maturation (Santonocito et al., 2014; Qasemi and Amidi, 2020). We have also previously demonstrated that EV-associated miRNAs within FFs contribute to the highly coordinated signaling occurring between follicular cells necessary for the development of a competent oocyte (Gad et al., 2024; Sohel et al., 2013). Furthermore, EVs (Menjivar et al., 2023) carrying miRNAs (Pavani et al., 2022) are readily internalized by oocytes and embryos, and FF-derived EVs have been shown to promote cumulus expansion—both morphologically and at the level of gene expression—*in vitro* (Hung et al., 2015). Building on these findings, the present study aimed to characterize, for the first time in the SWR, the transcriptomic differences in GCs collected *in vivo* from follicles that resulted in oocytes matured *in vitro* (PB) or that did not (NoPB) and to investigate the miRNA profiles of FF-EVs and GCs of the two sample groups. The later comparison can be useful in many ways, including identifying potential miRNA markers in FF or GCs as to whether the oocyte contained within that follicle has the potential to mature *in vitro* and in identifying miRNAs that could be used to supplement the *in vitro* maturation system, aiding in the maturation of a competent oocyte suitable for fertilization. The authors recognize that the sample number is low for this study due to the nature and rarity of these samples, but the power of the study is increased because the same GCs were used for both the mRNA and miRNA analysis, as well as the EVs came from the FF of a single follicle containing the corresponding GCs.

Overall, in our transcriptome analysis of GCs from the two follicle categories (PB and NoPB) revealed that there were 1,117 transcripts differentially expressed between groups. This resulted specifically in 207 annotated genes, four of which had multiple transcripts (*CARS2, DNAAF11, GFM2,* and *SLC22A13*). Interestingly, for *SLC22A13* and *DNAAF11,* the two transcripts for each gene had opposite expression patterns (i.e., one transcript was upregulated in PB and the other in NoPB). *SLC22A13* has been indicated as a crucial factor in NAD + replenishment, necessary for developmental competence in maturating oocytes (Liang et al., 2023). To date, there is no knowledge on the role of *DNAAF11* in oocyte maturation, but the variable transcript expression between these genes could indicate the crucial role of miRNA expression in determining the correct transcript splice sites. As noted in Figure 3, the primary targets of these genes included ubiquitin events such as ubiquitin-mediated proteolysis, protein ubiquitination, protein polyubiquitination, and proteosome-mediated ubiquitin-dependent protein catabolic process. Post-translational modification of cellular proteins by ubiquitin and ubiquitin-like modifiers represents a critical regulatory mechanism in gamete and embryo physiology, influencing key processes such as oocyte maturation, fertilization, and embryonic development through term (Mtango et al., 2014).

Within the collected GCs, there were 11 DE-miRNAs identified (Table 4), and all were upregulated in the PB group. This implies that this set of miRNAs may target the genes needed to be downregulated for meiotic competence to occur. Two of these miRNAs were found in multiple species. miR-1307, while identified in horse and in human in our dataset, has also been identified in sows to be associated with low reproductive efficiency (Hu et al., 2020). The other miRNA identified in our dataset in multiple species was miR-125b-5p. Specifically in the sheep, this miRNA has been identified to be associated with early pregnancy (Gözer et al., 2025). In addition, miR-125b-5p has been associated with follicular development (Zhang et al., 2025). These studies indicate that while our dataset identified these miRNAs in multiple species, additional literature supports that finding and their role in oocyte development. The gene targets for these eleven DE-miRNAs were also identified. Of these, six of the miRNAs directly target DEGs from the GCs transcriptome dataset. miR-194-3p potentially targets three genes: *DEPDC1, GJB4, and TK1*. Noteably, *DEPDC1* (DEP domain-containing protein 1) and *GJP4* (Gap Junction Protein Gamma 4) were both upregulated in the NoPB group. A previous study demsontrated that *DEPDC1* is a key regulator in cell cycle progression and meiosis (Mi et al., 2015; Feng et al., 2017). More specifically, *DEPDC1* is associated with the progesterone-mediated oocyte maturation pathway (Wu et al., 2019) and its decreased levels are known to cause cell arrest (Mi et al., 2015). The gene *GJP4* has been reported to play a pivotal role in communication between the oocyte and the surrounding cells (Kidder and Mhawi, 2002; Eppig et al., 2002). Gap junctions allow the flow of cAMP and cGMP, which keep the oocyte arrested in prophase I (Eppig et al., 2002). In this dataset, *GJP4* was found exclusively expressed in the GCs from the NoPB group, indicating this could have been an indicator of improper meiotic arrest in these oocytes and result in maturation failure. *TK1* (thymidine kinase 1), a key enzyme in the DNA salvage pathway, is primarily associated with cell proliferation and nucleotide metabolism, and while not directly involved in oocyte meiotic maturation, its expression in surrounding follicular cells may indirectly reflect conditions that support oocyte development (Sherley and Kelly, 1988; Eppig, 1996; Eppig, 2001). These results provide evidence of an interplay between the SWR GCs miRNA and mRNA expression patterns, highlighting a potential post-transcriptional regulatory mechanism governing GC function within the follicular microenvironment.

The final category of miRNAs evaluated comprised those contained within FF-EVs surrounding the GCs and oocytes. Of the 15 miRNAs identified to be DE, nine were highly expressed in the PB group, and six were highly expressed in the NoPB group. Interestingly, one of the highly expressed miRNAs in the NoPB group (miR-204-5p) was found to target 19 different DEGs identified in the GCs gene expression dataset. The primary gene of interest with a direct link to oocyte maturation was *IL-1β* (Interleukin-1 beta). This is a pro-inflammatory cytokine expressed in GCs and in cumulus cells present in the FF, participating in both paracrine and autocrine signaling, influencing steroidogenesis, follicular growth, and local immune regulation (Martoriati and Gérard, 2003). In animal studies, *IL-Iβ* promotes germinal vesicle breakdown (GVBD) in oocytes, a key step in meiotic resumption, and modulates cumulus cell function to enhance the production of prostaglandins and other factors that support meiotic progression (Martoriati and Gérard, 2003; Caillaud et al., 2005; Caillaud and Gérard, 2009). Mechanistically, *IL-1β* stimulates granulosa and cumulus cells, which then secrete signals such as progesterone, prostaglandins, and EGF-like ligands that trigger oocyte maturation; direct effects on oocytes remain unclear (Wang et al., 2002). In humans, FF *IL-1β* levels correlate with oocyte competence and embryo quality in IVF cycles, indicating it may serve as a biomarker of a healthy follicular environment, though a direct causal role has not been fully established (Zollner et al., 2013). So, higher expression of miR-204-5p in NoPB group may indicate a reduced expression of its target *IL-1β*, contributing to lower meiotic competence in those oocytes.

Another gene of interest targeted by miR-1246 (upregulated in PB) was *PCPB2* (poly(C)-binding protein 2). *PCBP2* has been implicated in ovarian follicle support, particularly through its role in GCs rather than its direct action on the oocyte. In a mouse model of premature ovarian insufficiency (POI), *PCBP2* expression was downregulated in ovaries and in cultured GCs exposed to cyclophosphamide. *PCBP2* stabilizes *IGF2* mRNA, promoting *IGF2* expression, which in turn reduces GC apoptosis, preserves mitochondrial function, and improves ovarian function, including increased ovarian weight and follicle numbers when *PCBP2* is overexpressed *in vivo*. We have previously identified higher expression of miR-1246 in GCs subjected to heat stress (Gebremedhn et al., 2020) and this miRNA was reported to negatively regulate *PCBP2* and cAMP response element binding protein-like 2 (*CREBL2*) mRNA and protein levels through binding to the 3′-UTR region and inhibited heat stress induced apoptosis in lung cells of cattle in lung cells (Hu et al., 2018).

When evaluating the miRNA, their targets, and the DEG expressed genes, a few genes stood out because they were targeted by multiple miRNAs, or by miRNAs from different sources. The first gene noted was *DEPDC1*, which as stated earlier, is associated with the progesterone-mediated oocyte maturation pathway in order for progression to occur (Wu et al., 2019). Overall, this gene is increased in the NoPB group, but it is being targeted by miR-194-3p found in the GC-miRNA (upregulated in the PB group) and miR-204-5p found in the FF-EVs (upregulated in the NoPB group). The canonical methodology for miRNAs is to downregulate their target gene (Valinezhad Orang et al., 2014), which would suggest these gene is being downregulated by the FF-EVs in the NoPB group, potentially inhibiting the oocyte from reaching meiotic competence. Another gene noted was *GJB4*, which as stated before, plays a pivotal role in communication between an oocyte and the surrounding cells (Kidder and Mhawi, 2002; Eppig et al., 2002). These gap junctions allow the flow of cAMP and cGMP, which keep the oocyte arrested in prophase I (Eppig et al., 2002). The same GC-miRNA targeting *DEPDC1* also targets *GJB4*, and it is also targeted by miR-637 found in the FF-EVs (upregulated in the NoPB group). *GJB4* is upregulated in the NoPB group, indicating this is a mechanism in place to prevent the oocyte from resuming meiosis. Interestingly, it appears the GC-miRNA is trying to decrease *GJB4* (to resume meiosis), but the FF-EVs are doing the opposite.

From this dataset, we suggest further evaluation of the miRNA miR-194-3p, as it affects multiple genes involved in the cell cycle (both resumption and halting of the cycle based upon which gene is being evaluated). This could be a potential biomarker to determine an oocyte’s ability to mature *in vitro*, especially because it is found to be increased in the GC surrounding oocytes that went on to mature during *in vitro* maturation.

In conclusion, this study identified miRNA in both FF-EVs and GCs that could be used as potential indicators of oocyte developmental competence following *in vitro* maturation. We provide a novel insight into the mechanistic actions occurring between the FFs and GCs *in vivo*, as amechanism to guide our applications *in vitro*. Additionally, potential interactions of EV–mediated molecular signaling during folliculogenesis in the SWR may offer critical insights into physiological and pathological reproductive conditions that contribute to infertility, ultimately providing biological information to drive reproductive strategies to enhance ART outcomes in this species and others.

## 5 Limitations and mitigation

The authors acknowledge that the sample number is low for this study compared to experimental numbers possible in domestic species. The samples we used for this study are collected in only a few places in the world due to the complexity of performing OPU in the white rhinoceros. We collected these samples opportunistically while focusing on collecting viable oocytes to put into maturation and fertilize for embryo development, the final goal of this project. We would like to emphasize that the cost, the work, and the challenges of performing a field procedure to collect these samples from such a large mammal is quite extraordinary. The authors feel privilidged do have had the opportunity to match this type advanced scientific approaches and analysis, easily possible in domestic species, in the white rhinoceros, a wildlife megafauna. In addition, these samples are from a single biological sample due to the difficulty in collections. To account for the low sample number, the power was increased by using the same GCs for both mRNA and miRNA analysis, as well as the extracellular vesicles came from the FF a single follicle containing the corresponding GCs.

The limited availability of species-specific genomic and miRNA annotations for the SWR represents an inherent limitation of this study. The reliance on proxy annotations from human, bovine, and equine datasets, while necessary for functional interpretation, may bias results toward conserved biological processes and obscure species-specific regulatory features. To address this, we implemented a genome-guided miRNA discovery approach, enabling the identification of novel miRNAs directly from the NWR genome, which represents the closest available reference but may not fully capture species-specific genomic features. Additionaly, we employed an integrated analytical framework combining GC mRNA and miRNA profiling with FF-EV miRNA analysis. This multi-compartment approach enabled cross-validation of molecular signals, allowing the identification of consistent and biologically relevant miRNA–mRNA interactions across independent data sources. The convergence of findings between GC and FF-EV datasets strengthens confidence in the observed regulatory networks and reduces the likelihood of dataset-specific artifacts.

## Data Availability

The datasets presented in this study can be found in online repositories. The names of the repository/repositories and accession number(s) can be found below: https://www.ncbi.nlm.nih.gov/, GSE300824 https://www.ncbi.nlm.nih.gov/, GSE326340.
